# Unlocking dynamics of goal-scoring: the showdown between direct and indirect transition goals across football leagues

**DOI:** 10.5114/biolsport.2025.142640

**Published:** 2024-09-25

**Authors:** Pedro Eusebio, Pablo Prieto-González, Rui Marcelino

**Affiliations:** 1 University of Maia, Portugal; 2 Research Centre in Sports Sciences Health Sciences and Human Development, CIDESD, CreativeLab Research Community, Vila Real, Portugal; 3 Sports Sciences and Diagnostics Research Group, GSD-HPE Department, Prince Sultan University, Riyadh 11586, Saudi Arabia; 4 Portugal Football School, Portuguese Football Federation, Oeiras, Portugal

**Keywords:** Performance analysis, Counterattacks, Offensive transition, Elite soccer, Game moments

## Abstract

Offensive transitions, (defined as shifts from defense to attack) have an extraordinary impact on goal scoring patterns. Identifying the factors that most significantly influence its achievement is crucial, enabling teams to tailor strategies to their specific characteristics and the particular demands of their championships. The study aims to provide analyzes of the separate and combined impacts of various variables on the success of offensive transitions and their outcomes. The sample comprised 1151 games from nine distinct countries, categorized into three league groups: Top, Marginal, and Emerging. These matches yielded 1649 goals, which were classified as either direct offensive transitions or offensive transitions resulting from positive outcomes (goals scored from set-piece situations following successful offensive transitions). The statistical method employed was binomial logistic regression. A total of 20 to 23% of goals scored by Offensive transitions resulted from positive outcomes. Offensive transitions play a pivotal role in competitive leagues, with 47% of all goals. Top leagues exhibit an even higher proportion (53%) of goals originating from offensive transitions, emphasizing the effectiveness of defensive pressure in specific areas and involving more passes and offensive combinations. All league groups highlighted the central zones of the defensive midfield as essential to initiating successful direct offensive transitions. In Emerging Leagues, they are more likely to succeed with three passes than with two passes. In Marginal leagues, the number of players involved and the passes are related to the starting area. The findings enhance understanding of offensive transition tactics in football for greater scoring impact.

## INTRODUCTION

Football, also referred to as “Association Football” or “Soccer,” is distinguished by its intricate nature, which render its observation and analysis challenging to objectify [1]. Despite prior characterizations of football as a dynamic, interactive, and uncontrollable phenomenon [2], the scrutiny of tactical team performance enables the elucidation of team dynamics [3], as well as the detailed examination of the style of play and the attributes that most precisely delineate a particular mode of playing.

Despite the necessity of accounting for variables such as player and team capabilities [4], pre-competition analysis provides coaches with the tools to forecast and formulate diverse strategic approaches for forthcoming matches [5]. This process entails the careful selection of particular combinations of offensive and defensive tactics, with a thorough assessment of their respective strengths and weaknesses, to optimize the likelihood of achieving success [6] and ultimately enhance overall performance.

Whether the game style is defined as “Transitional Game”, “Counter-Attack Game” [5, 7] or simply “Counterattack” [8], it is unequivocal that there is dominance in the moments of transitions (attack/defense or defense/attack). Also, those moments of transition are the ones that have high scoring opportunity rates [9–11]. While possession of the ball and a team’s ability to retain are often overvalued, there is non-consensus about the importance of maintaining possession for extended periods versus shorter possessions. Numerous authors have reported that possessions that enable fast and transitional attacks are the ones most likely to lead to success [5, 8, 12, 13].

Several studies have provided insights into goal-scoring strategies in different competitions. Analysis of the 2004 European Championship games revealed that 20.3% of goals were scored through counterattacks and 35.6% from set pieces [9]. Similar patterns were observed in the 2006 World Cup, where 20.3% of goals originated from counterattacks and 32.6% from set pieces [14]. During the knockout rounds of the 2010 World Cup, 18.8% of goals were attributed to counterattacks and 20.0% to set pieces [15]. However, research by Wright et al. [16] on the English FA Premier League demonstrated a different trend, with 35.6% of goals resulting from set pieces, but a notable 63% of all goals being scored through transitional play, a figure three times higher than those reported in the aforementioned studies. Eusebio et al. [17] recently highlighted all of these studies in their narrative review, underscoring the significance of transition moments in football and the importance of goals scored during these periods, as well as the versatility of the competitions in which they occur. Understanding whether these numbers differ by competition (leagues versus knockout competitions) and how offensive transitions contribute to the goals scored by set pieces is essential. Thus, it is pertinent to analyze the successful direct offensive transitions, but also, the goals obtained from positive outcomes of offensive transitions. The moments of play and processes are not independent from each other, meaning that a play comes as a consequence of the precedent play [17]. The link between plays reinforces the pathway of preceding decisions [18].

Positive outcome goals may be defined as those resulting from set pieces, originating in an offensive transition that culminates in a penalty kick, corner kick, free kick, or throw-in within the final third of the field. In this context, the play immediately preceding the goal must be an offensive transition. For instance, a goal scored from a penalty kick would typically be classified as a set-piece goal; however, if it follows an offensive transition, it will be categorized as a goal achieved through a Positive Outcome.

The effectiveness of “coordination tactics” in football teams has already been demonstrated [19]. Several other variables, (i.e., first action, recovering area, and number of passes) have been linked with the success of the transitions. Hewit et al. [20] explored the number of passes required to score a goal, building upon Pollard Reep’s [21] finding that 80% of goals arise from just three passes, albeit without specifying the attacking play involved. Additionally, Hughes and Franks’ [10] research on the 1990 FIFA World Cup revealed that successful teams with passing sequences exceeding five passes per possession produced more goals than those with shorter sequences. Also, the moment of possession loss presents a window of opportunity for the attacking team to initiate an offensive transition and potentially compromise the success of the opposing team’s defensive transition, leading to greater finishing opportunities [22].

Based on the findings outlined above, this study aims to further explore the factors influencing goals scored in offensive transitions. Additionally, the study seeks to examine both the individual and synergistic impacts of various variables across different league groups. The objective is to provide insights into the significance of direct offensive transitions and the successful outcomes of these transitions, elucidating their role in achieving successful outcomes. To enhance comprehension of the correlations between direct offensive transitions and their successful outcomes, various factors were investigated, including the initial action, the number of participating players, the number of passes, the origin and destination zones, the transition length, the match venue, and the category of favorable outcomes that influence their occurrence. The ultimate goal was to enhance our understanding of offensive transitions in football and their contribution to goal-scoring, thereby contributing to advancing tactical knowledge in the sport.

## MATERIALS AND METHODS

### Sample and variables

This investigation utilized a sample of 1151 games from the 2019–20 season, encompassing the games played in the Emerging, Marginal, and Top Leagues analyzed from the season’s commencement until the midpoint, leading to 3497 goals. The leagues were grouped into three groups based on the UEFA ranking and the latitude of each league. The Top Leagues group consist of the Spanish (La Liga), Italian (Serie A), and German leagues (Bundesliga), which were classified as 2^nd^, 3^rd^ and 4^th^ in the UEFA ranking (at the date the events were collected). These leagues have the highest turnover and value invested in the transfer markets. The Marginal leagues, consisting of the Portuguese (Primeira Liga), Dutch (Eredivisie), and Russian (Premier League) leagues, which are ranked 6^th^, 7^th^, and 8^th^ respectively and present a high number of transfers to and from others analyzed leagues. The Emerging Leagues (EL) are made up of the Qatar (Stars League), Saudi Arabia (Pro League), and UAE (Pro League) leagues which appear as markets with high financial potential that manage to attract some international players but those leagues are played mainly by local players.

**FIG. 1:**
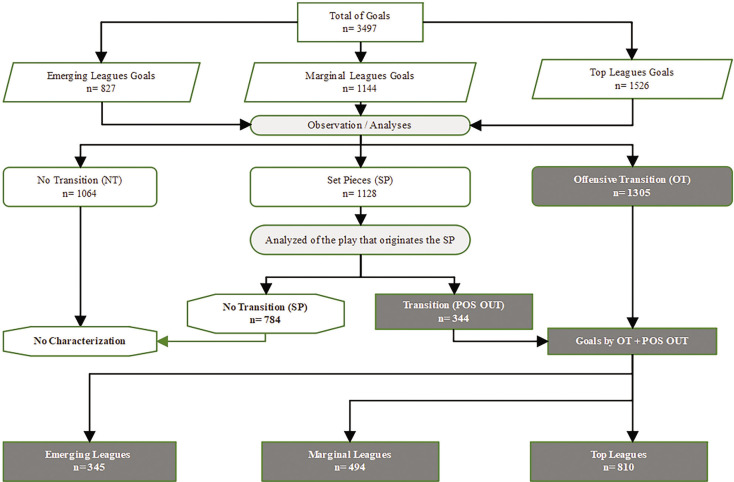
Sample algorithm Note: All goals in the nine leagues are analyzed and classified accordingly. The goals that result from Direct Offensive transition (OT), as well as those that result from a Positive Outcome (POS OUT), will be characterized and described in detail and will represent the sample. All others do not constitute the sample.

All goals were categorized into three types: i) Non-offensive Transition (NT), ii) Direct Offensive Transition (OT), and iii) Set Pieces (SP). The goals that fell into Set Pieces were analyzed based on the play immediately preceding the goal. If the play that immediately preceded the goal was not a transition, the goal remains classified as Set Pieces; however, if that play was an offensive transition play, the goal was classified as a Positive Outcome (POS OUT) (figure 1). A total of 1649 goals were scored due to direct offensive transitions or from positive outcomes.

### Procedures

**FIG. 2:**
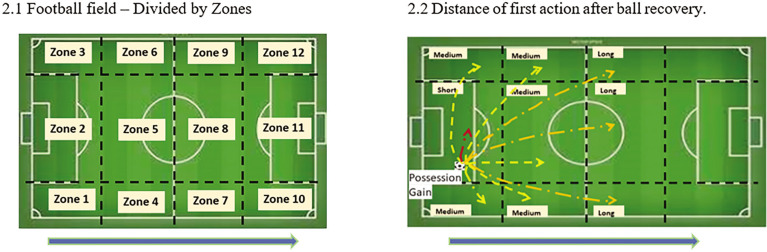
Variables decisions options Note: 2.1) The starting zone: area of the ball’s recovery. 2.2) Distance of the first action: Short if the first action ends in the same area as started; Medium if the first action finishes in the near zone; Long if the first action ends in a zone far from the beginning of the play.

The instrument used for the analysis was adapted from the system proposed by Turner and Sayers [11], along with an observation and registration instrument proposed by the authors, which is constituted by systems of categories that precisely define the target registration criteria. The videos were obtained from two video providers, InStat and WyScout. To maintain methodological rigor and ensure comparability across teams, only games with goals from the first round to the midway point of each championship were included in the analysis. As a result, all teams were subject to an equivalent number of observations, and every team in each championship played with each other. All plays that resulted in a goal were analyzed from the moment the ball was recovered until the goal was scored. This includes scenarios where a team gains or regains ball control through a tackle, interception, or rebound. In cases of quick restart of the game that led to an offensive transition, the moment of the beginning of the first action, such as a throw-in, goal kick, or free kick, was considered. The goals scored by Offensive Transition (OT) were meticulously described, focusing on the following variables: i) The first action taken after possession is gained: pass or drive; ii) The number of players involved: the number of players that touched the ball; iii) The number of passes made: the number of passes during the transition; iv) The starting zone: the area of ball recovery; v) The distance of the first action: the distance of the first pass/drive; vi) The Match status (home/away): whether the team was playing at home or away; vii) The type of positive outcome: corner kick, free kick, throw-in, penalty kick; viii) The chronology of the goals. The goals classified as Positive Outcome (POS OUT) were considered as Offensive Transition (OT), with the end of the play defined as the moment of the positive outcome. Figure 2 provides a visual representation of the algorithm for the various options considered for these variables.

Five researchers underwent initial training sessions spanning 8 hours over 3 days to observe and analyze specific events. Subsequently, they independently conducted observations and codifications using a standardized instrument. Subsequently, the researchers were provided with 30 events to analyze and code independently. After three weeks from the initial training, the analysis of events was revisited, with only those researchers demonstrating homogeneous observation and analysis criteria among themselves continuing in the study. This refinement left three researchers. This iterative process was repeated a second time until consensus was reached among all observers and analysts.

### Statistical analysis

The assumptions of normality and heterogeneity were verified with the Kolmogorov-Smirnov and Levene tests, respectively. Following this verification multiple Binomial Logistic Regression analyses were conducted to investigate the association between the dependent variable Goals by Direct Transition (OT),” and “Indirect Transition (POS OUT)” and the independent variables (see table 1).

**TABLE 1:** Independent variables and their respective categories analyzed with respect the dependent variable Goals by Direct Transition (OT) and Indirect Transition (POS OUT)

Independent Variable	Category
The first action taken after possession is gained	Pass or Drive
The number of players involved	1 to 11
The number of passes made during the transition	0 to +12
The starting zone of the transition	Z1 to Z12 (See fig 2.1)
Distance of the first action	Short Medium Long (See fig 2.2)
The Match status (home/away)	Home or away;
Type of Positive outcome	Corner Kick, Free Kick, Throw-In, Penalty Kick
Chronology of the goals	1st to 10th

On the occasions where associations were identified among these variables, the findings were recorded and subsequently aggregated in the Adjusted model to investigate the persistence and magnitude of these associations. This adjustment aimed to ascertain the robustness of the observed associations and understand the extent to which they maintained significance. The adjusted model allowed for a more nuanced exploration of the relationships between the independent variables and the specified goal transition types. The aim was to identify categories and the predictors that influence the likelihood of scoring goals through Offensive Transitions (OT) and Positive Outcomes (POS OUT) in each group of leagues. All the analyses were performed by Jamovi – Software.

### Ethics approval and consent to participate

All the video footage used is publicly available. Thus, no informed consent or ethics committee approval was required [23, 24]. Also, since the analyzed data was retrospective, the athletes were performing during their competitive season, while images of the games were broadcast on free-to-air TV. Ethics Methods Committee clearance was not required [25]. By informing all participating players, all tracking complies with the general data protection regulation (GDPR) https://gdpr-info.eu/, accessed 07/20/20. Nevertheless, the research received approval from the Ethics Committee of the University of Maia (37/2021) and was conducted in compliance with the guidelines of the Declaration of Helsinki.

## RESULTS

### Descriptive Analysis

In the 1151 games analyzed (as shown in Table 2), 47% of all goals (1649) were scored through direct offensive transitions resulting in positive outcomes. This pattern is particularly notable in Top leagues, where 53% of goals originate from such transitions, followed by marginal leagues at 43%, and Emerging leagues at 42%. A detailed breakdown of the results for each league is available in Table S1.

**TABLE 2:** Distribution of the types of goals per leagues

	Games Observed with Goals	Total Goals	Goals by NT + SP	Goals by OT + POS OUT	% Of goals By OT + POS OUT
**Top Leagues**	498	1526	716	810	53.08%
**Marginal Leagues**	391	1144	650	494	43.18%
**Emerging Leagues**	262	827	482	345	41.72%
**Totals**	1151	3497	1064	1649	47.15%

Note: Top Leagues: Germany, Spain, and Italy Leagues; Marginal Leagues: Netherlands, Portugal, and Russia leagues; Emerging Leagues: UAE, Qatar, and Saudi Arabia Leagues.

NT: Goals by No Transition; SP: Goals by Set Pieces; OT: Goals by Offensive Transitions (direct); POS OUT: Goals by Positive Outcomes; NT + SP – Goals where there is no offensive transition reported (direct or Positive outcome). Goals from Non-transitions and Set Pieces. OT + POS OUT – Goals obtained by Offensive transition and Positive Outcome.

As for the percentages and nominal values obtained in the different groups of leagues for each of the observed variables, direct offensive transitions account for 79% of the goals in the Top Leagues, 76% in the Emerging Leagues, and 80% in the Marginal leagues (see Table 3).

**TABLE 3:** Distribution of the OT+POS OUT goals, relative frequencies of the studied variables ascross the three groups classified.

Performance indicators	Top Leagues % (n = 810)	Marginal Leagues % (n = 494)	Emerging Leagues % (n = 345)	Performance indicators	Top Leagues % (n = 810)	Marginal Leagues % (n = 494)	Emerging Leagues % (n = 345)
**Goals by Transition**				**Match Status**			
**OT**	**79.38** (n = 643)	**80.36** (n = 397)	**76.81** (n = 265)	**Home**	**52.72** (n = 427)	**54.66** (n = 270)	**52.75** (n = 182)
**POS OUT**	**20.62** (n = 167)	**19.64** (n = 97)	**23.19** (n = 80)	**Away**	**47.28** (n = 383)	**45.34** (n = 224)	**47.25** (n = 163)
**Zone of 1^st^ action**			**1^st^ Action**			
**Short**	**41.60** (n = 337)	**40.89** (n = 202)	**38.84** (n = 134)	**Pass**	**75.92** (n = 615)	**68.42** (n = 338)	**76.52** (n = 264)
**Medium**	**51.60** (n = 418)	**50.40** (n = 249)	**52.17** (n = 180)	**Drive**	**24.07** (n = 195)	**31.58** (n = 156)	**23.48** (n = 81)
**Long**	**6.79** (n = 55)	**8.70** (n = 43)	**8.99** (n = 31)			

**Goal by transition Nr**				**Nr players involved**			
**1**	**32.22** (n = 261)	**33.40** (n = 165)	**29.57** (n = 102)	**1**	**5.93** (n = 48)	**7.69** (n = 38)	**4.06** (n = 14)
**2**	**26.67** (n = 216)	**26.11** (n = 129)	**23.19** (n = 80)	**2**	**16.67** (n = 135)	**18.62** (n = 92)	**19.71** (n = 68)
**3**	**20.37** (n = 165)	**18.42** (n = 91)	**21.16** (n = 73)	**3**	**26.06** (n = 211)	**30.57** (n = 151)	**32.75** (n = 113)
**4**	**11.36** (n = 92)	**12.35** (n = 61)	**16.23** (n = 56)	**4**	**25.06** (n = 203)	**22.67** (n = 112)	**25.80** (n = 89)
**5**	**5.31** (n = 43)	**6.68** (n = 33)	**5.51** (n = 19)	**5**	**15.31** (n = 124)	**12.75** (n = 63)	**12.17** (n = 42)
**6**	**2.84** (n = 23)	**2.63** (n = 13)	**2.31** (n = 8)	**6**	**6.91** (n = 56)	**4.25** (n = 21)	**3.48** (n = 12)
**7**	**0.99** (n = 8)	**0.40** (n = 2)	**1.74** (n = 6)	**7**	**2.35** (n = 19)	**2.23** (n = 11)	**1.16** (n = 4)
**8**	**0.12** (n = 2)	**0.00** (n = 0)	**0.29** (n = 1)	**8**	**0.99** (n = 8)	**0.81** (n = 4)	**0.87** (n = 3)
**Nr of Passes**				**9**	**0.62** (n = 5)	**0.20** (n = 1)	**0.00** (n = 0)
**0**	**6.17** (n = 50)	**7.49** (n = 37)	**3.77** (n = 13)	**10**	**0.12** (n = 1)	**0.20** (n = 1)	**0.00** (n = 0)

**1**	**16.67** (n = 135)	**17.00** (n = 84)	**17.68** (n = 61)	**Start Zone**			
**2**	**20.49** (n = 166)	**24.49** (n = 121)	**26.38** (n = 91)	**Z1**	**2.59** (n = 21)	**4.86** (n = 24)	**3.48** (n = 12)
**3**	**21.35** (n = 173)	**21.26** (n = 105)	**26.67** (n = 92)	**Z2**	**23.09** (n = 187)	**23.48** (n = 116)	**25.22** (n = 87)
**4**	**17.65** (n = 143)	**17.21** (n = 85)	**14.78** (n = 51)	**Z3**	**3.46** (n = 28)	**2.43** (n = 12)	**3.19** (n = 11)
**5**	**8.77** (n = 71)	**5.47** (n = 27)	**6.67** (n = 23)	**Z4**	**7.28** (n = 59)	**7.09** (n = 35)	**8.12** (n = 28)
**6**	**4.20** (n = 34)	**2.83** (n = 14)	**2.03** (n = 7)	**Z5**	**16.30** (n = 132)	**16.19** (n = 80)	**17.97** (n = 62)
**7**	**2.22** (n = 18)	**2.02** (n = 10)	**1.16** (n = 4)	**Z6**	**7.04** (n = 57)	**7.69** (n = 38)	**7.25** (n = 25)
**8**	**1.23** (n = 10)	**0.81** (n = 4)	**0.29** (n = 1)	**Z7**	**6.67** (n = 54)	**6.07** (n = 30)	**5.22** (n = 18)
**9**	**0.25** (n = 2)	**0.20** (n = 1)	**0.29** (n = 1)	**Z8**	**16.30** (n = 132)	**16.60** (n = 82)	**11.59** (n = 40)
**10**	**0.25** (n = 2)	**0.40** (n = 2)	**0.29** (n = 1)	**Z9**	**5.56** (n = 45)	**6.68** (n = 33)	**7.24** (n = 25)
**11**	**0.37** (n = 3)	**0.20** (n = 1)	**0.00** (n = 0)	**Z10**	**2.35** (n = 19)	**1.62** (n = 8)	**2.90** (n = 10)
**12**	**0.12** (n = 1)	**0.00** (n = 0)	**0.00** (n = 0)	**Z11**	**7.78** (n = 63)	**6.28** (n = 31)	**6.67** (n = 23)
**POS OUT**				**Z12**	**1.60** (n = 13)	**1.01** (n = 5)	**1.16** (n = 4)

**Corner Kick**	**29.94** (n = 50)	**28.87** (n = 28)	**31.25** (n = 25)				
**Goal Kick**	**0.00** (n = 0)	**0.00** (n = 0)	**0.00** (n = 0)				
**Throw in**	**2.99** (n = 5)	**5.15** (n = 5)	**2.50** (n = 2)				
**Free Kick**	**24.55** (n = 41)	**25.77** (n = 25)	**27.50** (n = 22)				
**Penalty Kick**	**37.13** (n = 62)	**40.20** (n = 39)	**38.75** (n = 31)				

Due to the relevance constraints of space, only the results demonstrating relevant associations are presented herein. Thus, the predicted variables found were i) Start Zone in the Top Leagues, ii) Start Zone, Nr of Players involved and Nr of Passes in the Marginal Leagues, and iii) 1^st^ Action and Number of Passes in the Emerging Leagues. Those associations are presented in tables 4, 5, and 6 within the Top Leagues, Marginal Leagues, and Emerging leagues, respectively. The remaining outcomes are omitted as they did not demonstrate predictive capacity.

As an illustration, within the emerging leagues (table 6), transitions instigated through a passing action exhibit a 1.995-fold higher likelihood of culminating in a Direct Transition (OT) as opposed to an Indirect Transition (POS OUT), in comparison to transitions instigated through a driving maneuver, particularly when evaluating transitions that lead to a goal.

In the top leagues, it was observed that the “Starting Zone” variable could predict the variability of direct Transitions off Indirect Transitions (Table 4). Since the identified association between goals scored through direct and indirect transitions within a single variable, there was no need to perform the adjusted model.

**TABLE 4:** Binomial logistic regression of the performance indicators associated with Goals by Direct Transition (OT) vs Indirect Transition (POS OUT) – Top Leagues

Goals by Direct Transition (OT) vs Indirect Transition (POS OUT) – Top Leagues		

Binomial logistic regression		

Performance indicators	p	OR (95% CI)
**Start Zone**		
*Z5 vs Z7*	0.017	3.116 (1.23–7.90)
*Z12 vs Z7*	0.025	5.000 (1.23–20.34)
*Z2 vs Z11*	0.021	3.157 (1.19–8.39)
*Z3 vs Z11*	0.034	3.867 (1.11–13.52)
*Z5 vs Z11*	0.003	4.518 (1.68–12.15)
*Z6 vs Z11*	0.029	3.427 (1.14–10.33)
*Z8 vs Z11*	0.013	3.560 (1.31–9.66)
*Z12 vs Z11*	0.007	7.250 (1.71–30.70)

Note: *p: p* value; OR: Odd ratios; 95% CI: confidence intervals (95%);

Regarding the Marginal Leagues, several factors were significantly associated with the goals scored by different types of transitions (table 5).

**TABLE 5:** Binomial logistic regression of the performance indicators associated with Goals by Direct Transition (OT) vs Indirect Transition (POS OUT) – Marginal Leagues

Goals by Direct Transition (OT) vs Indirect Transition (POS OUT) – Marginal Leagues				

	Binomial logistic regression		Adjusted Model	

Performance indicators	p	OR (95% CI)	p	OR (95% CI)
**Start Zone**				
*Z1 vs Z11*	–	–	0.028	8,742 (1.27–60.24)
*Z2 vs Z11*	–	–	0.005	10,237 (2.05–51.21)
*Z5 vs Z11*	0.027	5,500 (1.21–25.01)	0.004	10,864 (2.18–54.08)
*Z6 vs Z11*	–	–	0.025	7,226 (1.28–40.91)
*Z7 vs Z11*	–	–	0.042	6,241 (1.07–36.39)
*Z2 vs Z8*	–	–	0.019	2,671 (1.17–6.09)
*Z5 vs Z8*	–	–	0.014	2.831 (1.23–6.63)

**Nr Players Involved**				
*1 vs 4*	0.001	4.333 (1.76–10.65)	–	–
*3 vs 4*	0.011	2.514 (1.24–5.11)	–	–

**Nr of Passes**				
*0 vs 3*	0.028	2.750 (1.12–6.78)	–	–
*2 vs 3*	0.008	2.540 (1.28–5.06)	–	–
*7 vs 3*	0.038	4.333 (1.09–17.31)	–	–
*0 vs 4*	0.019	3.173 (1.21–8.33)	–	–
*2 vs 4*	0.006	2.930 (1.36–6.33)	0.028	4.131 (1.17–14.59)
*7 vs 4*	0.027	5.000 (1.20–20.83)	0.024	6.787 (1.29–35.60)
*0 vs 5*	0.027	5.000 (1.20–20.83)	–	–
*7 vs 5*	–	–	0.048	6.573 (1.01–42.60)

Note: *p: p* value; OR: Odd ratios; 95% CI: confidence intervals (95%);

**TABLE 6:** Binomial logistic regression of the performance indicators associated with Goals by Direct Transition (OT) vs Indirect Transition (POS OUT) – Emerging Leagues

Goals by Direct Transition (OT) vs Indirect Transition (POS OUT) – Emerging Leagues				

	Binomial logistic regression		Adjusted Model	
**Performance indicators**	p	OR (95% CI)	p	OR (95% CI)

**1^st^ action**				

*Pass vs Drive*	0.044	1.995 (1.02–3.91)	–	–

**Nr of Passes**				

*3 vs 2*	0.030	2.158 (1.08–4.33)	0.043	2.058 (1.02–4.15)

Note: *p: p* value; OR: Odd ratios; 95% CI: confidence intervals (95%).

## DISCUSSION

The objective of the present investigation was to examine the separate and synergistic impacts of diverse factors, such as the initial action, number of participating players, number of passes, origin and destination zones, transition length, match venue, and favorable outcome category, that determine the goals scored in the context of direct offensive transitions, as opposed to those achieved through successful indirect transitions. The variables scrutinized in these offensive transitions include the initial action taken after gaining possession, the number of participating players, the number of passes executed, the origin and destination zones, the duration of the transition, the venue of the match, and the category of favorable outcome. Forty-seven percent of total goals in all group leagues originated from these moments, with the proportion increasing to over 53% in the top leagues (Table 2). These findings underscore the significant impact of transitions in the most competitive leagues, affirming that transitions are a crucial phase for disrupting the balance between teams in contemporary football [26].

The study emphasizes the relevancy of including goals resulting from positive outcomes of offensive transitions as part of the overall goals scored from offensive transitions. These goals constitute a substantial percentage across different leagues: 21% in top leagues, 20% in marginal leagues, and 23% in emerging leagues. This inclusion underscores their significance, especially given that currently, over 20% of transition goals are not recognized as originating from offensive transitions. The impact of these goals on overall goal-scoring surpasses conventional literature perceptions, which typically state that goals from transitions account for 18% to 20% of total goals [9, 14, 15].

All group leagues exhibit a pattern where successful offensive transitions (OT+POS OUT) predominantly stem from central zones of the field (Z2, Z5, and Z8) (Table 3). This suggests that turnovers in central areas compromise defensive recovery, making the team more susceptible to conceding goals [27]. These results demonstrate that transitions are integral to a team’s offensive and defensive processes, even if they can occur independently of ball possession. Consequently, within a collective framework, certain players may find themselves in distinct moments of the game compared to others, thereby making it more effective to achieve success through offensive transitions [17]. Also, the fact that all league groups exhibit higher percentages of successful transitions when initiated in the defensive midfield raises questions about the efficacy and capability of teams to execute high-quality counter-pressing that enables rapid ball recovery. Although many teams consider this dynamic important, neither objective evidence of its efficiency nor an analysis of its application in top leagues is presented in the literature [28].

In the top leagues, an association was found between the starting zone of the offensive transition and the type of successful transition. Thus, the probability of scoring a goal through a direct offensive transition is substantially lower when starting in zone Z11 than in zones Z2, Z3, Z5, Z6, Z8, and Z12. These findings differ from the conclusions drawn by Garganta and collaborators [29] and Larson [30], who suggested that recovering the ball closer to the opponent’s goal increased the likelihood of scoring. Additionally, it should be noted that direct offensive transitions initiated in zones Z5 and Z12 predict greater success than those initiated in zone Z7. This implies that successful transitions require offensive space to exploit [31], whether through off-the-ball runs that displace and create doubts in the team transitioning defensively or through passes and even ball penetration. Defensive decision-making is hindered by the speed of events and the necessity to make decisions at that moment, often at a numerical disadvantage compared to the attacking side [11, 31, 32].

The data presented in this study highlight the increasing effectiveness of defensive pressure applied in the lateral areas of the final third of the field, particularly in zone 12. This suggests that defensive teams often target the offensive build-up play, which has recently gained popularity. Like the side zones in the final third, zone 8 represents an opportunity for action as it is less occupied by teams attempting to create their organized attacks. Therefore, despite being a crucial game region, it is often less crowded as players try to spread out and cover a larger portion of the field. This dispersion and reduced mutual support among players create favorable conditions for the defensive team to exploit opportunities and initiate offensive transitions with higher chances of success, taking advantage of the defensive disorganization of the team that lost possession. Furthermore, it is noteworthy that only 7% of successful transitions involve long actions in their first movement. This indicates that modern football transitions involve more players and rely on intricate combinations between teammates. This trend reflects the emphasis on teamwork, coordination, associations, and the utilization of various players to facilitate successful transitions [20].

In marginal leagues, the associations observed pertain to the area where offensive transitions (OT+POS OUT) occur, the number of players involved, and the number of passes executed to achieve success. The probability of scoring a goal through a direct offensive transition is higher when initiated in the defensive midfield (zones Z1, Z2, Z5, and Z6) than Z11 (central zone of the last offensive third). The results suggest an important relationship between the distance of the transition start and the number of passes required for its execution [11]. Also, in line with the top leagues the number of successful offensive transitions initiated in the most forward zone, occurs less frequently which is in opposition to the results obtained by Garganta and collaborators [29] and Larson [30]. Additionally, the possession recovered in this zone provides less space for a deeper and more expansive style of play since the goalkeeper can neutralize these plays. Furthermore, the fact that successful transitions predominantly start in the defensive midfield of the defending team reveals a lack of tactical maturity in defensive coverage, and lesser control of ball possession. This raises questions about the significance and the benefit of maintaining ball possession for extended periods. The debate on whether to maintain possession of the ball for extended periods has grown in the literature, with no consensus currently established. Additionally, the data derived from the adjusted Binomial Logistic Regression model for Z2 and Z5 (located in the defensive midfield) compared to Z8 (offensive midfield) indicate a greater likelihood of successful offensive transitions when initiated in the defensive midfield. Despite the common assumption that offensive transitions are extremely fast and exhaustive [29], these findings demonstrate that teams in these leagues can achieve high effectiveness even when engaging in a many passes and player interactions. This suggests that successful transitions initiated far from the goal do not necessarily require an extremely vertical and fastpaced approach. Instead, transitions commencing in the defensive midfield allow for increased interactions among players within the same team. These results align with the studies that show “no significant relationship between transition and speed” [11].

Furthermore, the central zones of the field are particularly relevant for initiating successful offensive transitions (OT+POS OUT). For example, Z2 (the defensive zone closest to the team’s own goal) accounts for 24% of successful transitions out of the total 62% initiated in the defensive midfield (as presented in table 3). Notably, transitions starting at Z8 (at the beginning of the offensive midfield) represent 17% of the successful transitions. These findings align with the results obtained from the adjusted Binomial Logistic Regression model, which indicates that direct offensive transitions are four times more likely to succeed when two passes are executed rather than four passes. Since each offensive transition corresponds to a defensive transition by the opposing team, the reduced number of passes required suggests deficiencies in the opponent’s defensive positioning and a lack of concentration around the ball carrier/loser at the onset of the transition, thus not necessitating the creation of imbalance through excessive passing. Considering the data presented in table 3, where 40% of transitions see their first action completed within the same area of possession recovery, it can be inferred that offensive transitions occur with players in close proximity, with decision-making being a crucial factor for success [33]. The long passes are used as the first action only in 9% of the successful offensive transition, indicating more neutral and secure passes than the disrupted long balls.

In Emerging Leagues, successful transitions most commonly started from the central zones of the field (Z2, Z5, and Z8) (table 3). The adapted model derived from the Binomial Logistic Regression analysis indicates that the defensive midfield had the highest frequency of transition initiations (Z2 and Z5), which explains the obtained results. This indicates that teams tactically displace themselves when possessing the ball in the offensive midfield, inadvertently taking risks without considering the preventive defensive tactical positioning for coverage [13]. This demonstrates a positional lack of control and tactical immaturity. The fact that successful offensive transitions begin in Zone 2 (Z2), closer to their own goal, suggests that the team in possession of the ball takes unnecessary risks regarding their tactical position, thereby allowing more space for the opposing offensive transition.

Moreover, the model suggests that in direct offensive transitions, the probability of using three passes was twice as high as using only two. This finding can be attributed to the distance to the goal and the reduced density of opposing defensive players (in the defense position) during possession loss.

It is worth noting that 52% of the goals resulting from offensive transitions (OT+POS OUT) in these leagues have in their first action the change of zone from where the transition starts (to the zones next to), indicating that these actions are disruptive of the defensive organizations. Results showed that the primary action used to initiate offensive transitions was pass, which had twice the success rate in direct offensive transitions compared to offensive transitions from a positive outcome. However, the relevance of this action decreased in the adjusted model when the number of passes in a successful offensive transition was considered. This indicates that the success of the offensive transition in these leagues is independent of the action that initiates it. Acquiring insights into how teams execute transitions will contribute to the development of a unique and distinctive collective profile akin to a fingerprint [19], enabling the anticipation of gameplay actions.

Although Plakias et al. suggested that player characteristics or team strategies could influence goal-scoring patterns [34]. They demonstrated that regardless of players’ characteristics, team culture, coach attributes, or adopted style of play throughout a season, these factors are not determinative. Instead, offensive transitions occur consistently, with a clear pattern, underscoring their significant impact on the game [18].

## CONCLUSIONS

The study’s findings emphasize the importance of including goals from positive outcomes of offensive transitions into tactical analysis. These goals constitute a significant portion of the total goals scored in offensive transitions across all league groups. Almost half of all goals in all league groups, and over half in top leagues, originate from offensive transitions (OT+POS OUT), highlighting their impact on the competitive balance among teams. In Top Leagues, successful offensive transitions predominantly began in central zones of the field, with varying probabilities of scoring goals depending on the initiation zones. Conversely, in Marginal Leagues, commencing offensive transitions from defensive midfield zones increases the likelihood of scoring goals compared to starting in the central zone of the offensive third. Transitions from defensive midfield zones facilitate increased player interactions within the same team. In Emerging Leagues, successful transitions often start in central zones of the field, particularly from the defensive midfield. The number of passes involved in direct offensive transitions significantly influences their success rate. Additionally, transitions that alter zones from their starting points disrupt defensive organizations. These findings underscored the effectiveness of defensive pressure in lateral areas of the final third of the field. They emphasized the prevalence of intricate player combinations and teamwork in modern football transitions. Such insights into the dynamics and strategies of successful offensive transitions across different league groups contribute to a deeper understanding of football tactics and strategies to enhance team performance within these leagues.

This study presents limitations. Football is a dynamic sport with many variables that impact game outcomes. Another constraint arises from the subjectivity inherent in notational analysis, as it is challenging to conduct within the confines of the game space and relies solely on the images captured by television broadcasts. By focusing on a single season and specific leagues, the study’s findings may not apply to broader contexts or different seasons. The categorization of leagues into three groups based on UEFA rankings and financial indicators might not fully capture the diversity of football leagues worldwide. Additionally, while the study examines various variables related to offensive transitions, such as the first action after possession, the number of players involved, and starting zones, other factors (like player characteristics and team strategies) could also influence goalscoring patterns. Furthermore, this study’s findings may not apply to all football contexts, especially at the amateur level, where player tactics and skills differ from those in professional leagues. Therefore, future studies should address these limitations by considering sample size and scope, league selection, variable inclusion, and results’ external validity and generalizability.

## Practical Application

Teams should prioritize the analysis of goals obtained from positive results of offensive transitions along with the total goals scored in offensive transitions, as they represent an important part of the total goals in different leagues. Likewise, understanding zone preferences to initiate successful offensive transitions is crucial. In elite leagues, central areas of the field are more effective, while in less competitive leagues, starting from the defensive midfield areas leads to higher success rates. The number of passes and player interactions also significantly influence the success of offensive transitions. Teams should focus on combining players and teamwork to facilitate successful transitions, especially in emerging leagues where these interactions are crucial. Defensive pressure in lateral areas of the final third of the field can effectively disrupt offensive transitions, emphasizing the need for coaches to target these areas with defensive strategies to disrupt opponents’ offensive play. It is essential to adapt the tactical approach depending on the characteristics of the league. For example, focusing on the defensive midfield areas for offensive transitions in less competitive leagues can lead to higher success rates. Successful offensive transitions only sometimes require much passing. Teams can exploit defensive vulnerabilities by initiating quick transitions with fewer passes, especially when opponents show deficiencies in defensive positioning. Understanding opponent tactics and weaknesses in defensive organization can inform offensive transition strategies, allowing teams to exploit defensive zones and vulnerabilities to increase the probability of scoring goals. Strategic use of zone changes during offensive transitions can disrupt defensive organizations, allowing teams to strategically use zone changes to create imbalances and exploit defensive weaknesses. Thus, it is crucial to evaluate possession strategies based on offensive transition dynamics and consider alternative approaches based on league characteristics.

## Availability of data and materials

The datasets used and/or analyzed during the current study are available from the corresponding author on reasonable request. All data generated or analyzed during this study are included in this published article and its supplementary information files.

The data are not publicly available due to privacy.
